# Development of an Automated Pain Facial Expression Detection System for Sheep (*Ovis Aries*)

**DOI:** 10.3390/ani9040196

**Published:** 2019-04-25

**Authors:** Krista McLennan, Marwa Mahmoud

**Affiliations:** 1 Department of Biological Sciences, University of Chester, Parkgate Rd, Chester CH1 4BJ, UK; 2 Department of Computer Science and Technology, University of Cambridge, 15 JJ Thomson Avenue, Cambridge CB3 0FD, UK; marwa.mahmoud@cl.cam.ac.uk

**Keywords:** facial expression, sheep, computer automated systems, welfare, pain

## Abstract

**Simple Summary:**

Detecting signs of pain in sheep is a challenging problem, as they are a prey species and would usually try to hide any signs that they are unwell or injured. This means that treating ill or injured sheep and preventing any further spread of contagious diseases such as footrot can be slow. The recent development and publication of a Sheep Pain Facial Expression Scale (SPFES) has provided a tool to reliably detect pain in this species. However, due to the increase in intensification in farming and larger flock sizes being cared for by individual farmers, there is less time to spend monitoring sheep for changes in behaviour that may indicate illness or injury. Having an automated system that could detect changes in the facial expression of individual sheep would mean that farmers could receive information directly about particular individuals that need assessment. This would allow treatment to be provided in a timely and direct manner, reducing suffering. We have been developing the SPFES further in order for it to become an automated system. In this paper, we present our novel framework that integrates SPFES concepts with automatic facial expression analysis technologies.

**Abstract:**

The use of technology to optimize the production and management of each individual animal is becoming key to good farming. There is a need for the real-time systematic detection and control of disease in animals in order to limit the impact on animal welfare and food supply. Diseases such as footrot and mastitis cause significant pain in sheep, and so early detection is vital to ensuring effective treatment and preventing the spread across the flock. Facial expression scoring to assess pain in humans and non-humans is now well utilized, and the Sheep Pain Facial Expression Scale (SPFES) is a tool that can reliably detect pain in this species. The SPFES currently requires manual scoring, leaving it open to observer bias, and it is also time-consuming. The ability of a computer to automatically detect and direct a producer as to where assessment and treatment are needed would increase the chances of controlling the spread of disease. It would also aid in the prevention of resistance across the individual, farm, and landscape at both national and international levels. In this paper, we present our framework for an integrated novel system based on techniques originally applied for human facial expression recognition that could be implemented at the farm level. To the authors’ knowledge, this is the first time that this technology has been applied to sheep to assess pain.

## 1. Introduction

With the increasing global demand for meat and dairy products, it is estimated that by 2050 food production will need to increase by 25%–70% [1]. Producers are already under increasing pressure to produce large quantities of high-quality food, which has led to an increase in intensification. However, the number of producers has fallen, meaning large numbers of animals require care by a single person on a daily basis. This increases the workload for producers and places immense strain on the individual, which could lead to a reduction in health, safety, and welfare standards [2]. In addition, the public have growing concerns regarding the health and welfare of food-producing animals in current management systems [3]. Any procedures or diseases that may result in pain are major sources of concern for the public [4,5,6,7,8]. 

Diseases such as footrot and mastitis are a major source of pain in sheep, negatively impacting welfare and productivity. Footrot is a highly contagious disease that causes severe lameness [9], and was estimated in 2005 to cost the UK sheep industry £24.4 million annually [10]. Mastitis caused by pathogens such as *Staphylococcus aureus* and *Mannheimia haemolytica* [11] causes painful lesions within the teat canal [12], and in severe cases can cause death of the ewe. Early detection and subsequent treatment of such health problems is vital to getting animals back to full health as soon as possible, and for reducing the spread of disease [13]. Detecting early signs of disease, as well as the associated pain, is difficult as sheep are a prey species and do not overtly express any signs of weakness. This means that effective treatment and pain management is often inadequate, resulting in poor welfare [14,15,16,17,18]. With large numbers of animals becoming increasingly close in proximity with increasing intensification, there is a need for an integrated whole-system approach to detecting and controlling the spread of disease [19]. In turn, this will reduce the impact on the welfare and overall productivity of the flock. There is a need to develop novel technologies to aid in meeting these demands whilst still maintaining high standards. 

## 2. Precision Livestock Farming

Precision livestock farming (PLF) is the use of technology to optimize the production and management of each individual animal, offering tailored care from feeding and milking, to aiding producers in their daily tasks that require handling [20,21,22]. Many of these technologies can continuously monitor each individual, recording behaviour, productivity and current health status. These technologies may consist of biosensors or bio-imaging that are sensitive and systematic in their monitoring, which when integrated with knowledge of immunology and subclinical biomarkers can precisely direct the producer to where the administration of medicines to livestock is needed [23]. In addition, the algorithms within these systems are able to learn at the individual level, learning what is normal for each animal [2], ensuring that direct and individualized treatment can be applied. This will increase the chances of controlling the spread of disease as well as the prevention of resistance across the individual, farm and landscape at both national and international levels, helping to safeguard the UK’s food and medicine supply [24]. Recently developed technological systems have been designed to improve the health and welfare of species such as cattle, pigs and chickens by increasing the chances of early disease detection [25,26,27,28], but these systems have not yet been developed for sheep.

## 3. Facial Expression as a Pain Recognition Tool in Sheep

The recent development of the Sheep Pain Facial Expression Scale (SPFES) [29] by the first author has provided producers and veterinarians with a reliable and effective tool to help them recognize and assess pain in sheep. Facial expression is the measurement of changes in the face or groups of muscles known as “action units” to an emotional stimulus, and is likely to be an involuntary response to pain being experienced by an animal [30]. Facial expression is considered to be an honest signal of the intensity of the pain [31,32] as, in humans, it becomes increasingly difficult to “hide” the facial expression of pain [32], and faked pain is easily identified [33,34]. Additionally, facial expression has the ability to assess the temporal nature of pain [35,36], demonstrating whether there is a high degree of fluctuation, or if there is constant pain through long-term continuous assessment of the expression. This continued assessment and detailed analysis of the pain will allow observers to have a better understanding of the frequency and duration of the pain, enabling the development of a better pain-management strategy. 

Scoring the facial expression of pain live requires a human to be present, which can affect the expression of pain [37,38]. An automated system that allows the learning of individual facial expressions, and the subsequent detection of when expressions have changed suggesting possible disease presence, is vital to improving the screening process. A well-integrated automated system would remove any subjectivity of the assessment, ensuring consistency of pain estimation. Additionally, it would improve efficiency in care as it would not require the continued presence of an observer to assess these changes before and after treatment. Our proposed novel framework combines concepts from SPFES with automatic facial expression analysis to create a new application for technology to fully assess pain in sheep. This offers great opportunities for the sheep industry, placing them at the forefront of this technology.

## 4. Current Status of an Automated Pain Facial Expression Detection System for Sheep

**Figure 1:**
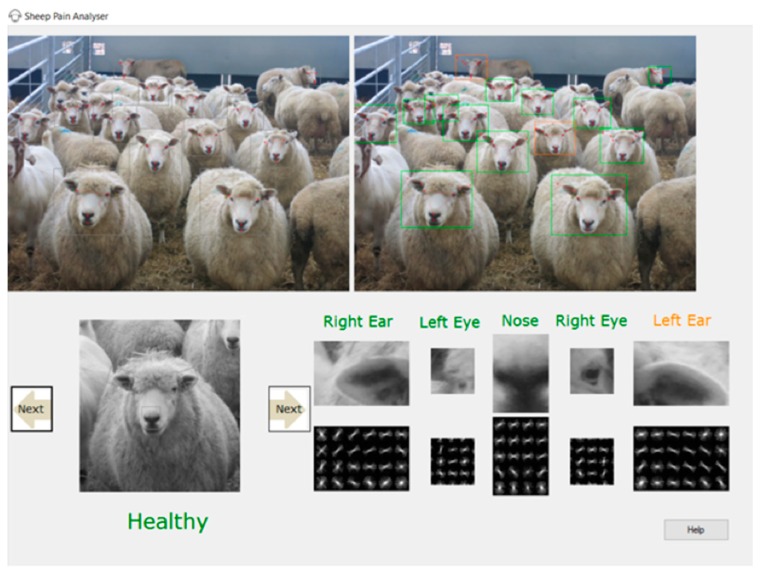
Pain analyser system prototype. **Top left**: photo of a flock of sheep, where sheep faces are automatically marked with boxes. **Top right**: facial expressions are automatically analysed using machine learning; animals with a probability of pain more than 50% are marked with an orange box while healthy animals are marked in green. **Bottom**: Every animal face is shown separately, and facial features are visualized, showing which areas in the face showed signs of pain. The final decision of the predictive model is based on the same scoring criteria of the Sheep Pain Facial Expression Scale (SPFES) scale.

Over the past two years, computer vision and machine learning methods have been used to commence the automatic detection of pain facial expressions in sheep. We modelled our approach based on techniques that are widely used in human facial expression recognition [39]. To the authors’ knowledge, this is the first time methods used in human facial expression recognition have been utilized to aid the development of an automatic system to assess pain in sheep. To automatically assess the signs of pain, an automated system needs to pass through a few stages: (1) detecting the face of the sheep in an image or video of the animal, (2) automatically localizing/marking important facial points/landmarks in the animal face representing major facial features (e.g., eyes, nostrils and mouth), (3) training machine learning models to learn changes in the facial features (action units) that indicate signs of pain based on the SPFES coding system, and (4) automatically assessing the pain score based on the machine learning models. It is possible to omit the third step and go directly to automatically assessing the pain level without the added step of automatically detecting changes in the action units. The full pipeline and the system prototype developed by the second author are presented in Figure 1. The technical details are described in more detail below.

Yang et al. [40] analysed sheep faces and proposed a novel approach using triplet-interpolated features in a cascaded shape regression framework to localize eight sparsely distributed facial landmarks as a first step to automate the process of facial expression analysis. Lu et al. [39] modelled changes in the localized facial landmarks to build an automatic system that can detect these changes as “action units”. The presented model extracted histograms of oriented gradients (HOGs) features from regions of interest around eyes, nose and mouth then trained a support vector machine (SVM) to estimate the “action unit” present based on the facial features. Based on the detected action units, a pain score was calculated based on the scoring criteria presented in the SPFES scale. A preliminary system describing the full pipeline is presented in Mahmoud et al. [41], which uses a basic face detection model that works mostly with frontal faces only. The challenge with the current sheep analysis model is that it requires frontal faces and does not consider facial changes in the cheeks and mouth of the sheep, as such changes are usually clearer in profile faces. In Hewitt and Mahmoud [42], a machine learning model based on deep learning localized 25 facial landmarks of the face of sheep and developed a model for handling profile faces as well as frontal faces. These results demonstrate that at this preliminary stage, the system can detect the face of individual sheep, in addition to detecting and assessing individual landmarks within the face and the corresponding pain scores—especially for frontal faces. Since all computer vision systems are based on building machine learning models, the availability of more labelled training datasets is key for future development in this area. 

## 5. Future Direction and Considerations

The use of technology to enhance the welfare of sheep is slowly increasing in relation to other production species; however, much of the progress made has been in research and development, and not in industry application [43,44]. We continue to develop the SPFES and its automated detection system with the aim of providing the sheep industry with the first novel technology that improves farm efficiency, productivity and animal welfare. There is still a wide area of improvement required, on both technical and practical levels, in order to reach a fully automated framework. The availability of labelled data is one of the main challenges, as the machine learning algorithms depend on a comprehensive set of reliably labelled datasets in order to build reliable models. We continue to work on the intermediate steps as well, including the reliable automatic detection of sheep faces (especially of different breeds), the automatic detection of changes in facial landmarks, and handling technical challenges such as lighting conditions and occlusion. We are presently adding to our current data set so that a variety of situations, breeds and farming systems are covered. There is a need to ensure that the technology recognizes the facial expression *change* and not simply facial expression, which would still require farm staff to assess and evaluate the facial expressions of sheep [43]. We have also planned usability studies in order to evaluate the efficiency of the automatic detection system compared to human coders. The system will then require matching with the current electronic identification device (EID) tags that all sheep in the UK must have. This will allow the producer to receive information about exactly which sheep they need to assess further. The practical logistics of these final steps will need careful collaboration with industry practitioners. 

The current system has been designed around assessing pain, but there are other affective states not yet recognized in sheep, and indeed in most other farm animals, such as stress, general malaise which may indicate illness, or even positive affective states such as joy. It is possible that our methodology could be applied to these other states, as well as to other species. Again, this would require significant effort to collect large data sets, and labelling of the states for the system to learn.

Additionally, in order to encourage uptake of the new technology onto farms, it must be able to integrate information from other on-animal sensors and external technologies. Combining data in this way would have significant benefits for the sheep industry. In particular, the early detection of diseases such as lameness and mastitis, or the detection of parturition, integrated with information about any changes in facial expression of the animal, would allow a much more rounded picture of the current state of the individual. Better treatment and prevention strategies could be developed as a result of such data sets. There will also be a need to demonstrate the economic and animal welfare benefits. The continued development and use of technologies will help to reduce the significant costs often associated with initial set-up and use of such systems. The uptake of this new technology in farms is more likely to occur when there are more data demonstrating its practical application to improving animal welfare and its benefit to farming.
